# Inactivation kinetics of *Cronobacter sakazakii* and *Salmonella enterica* in reconstituted powdered infant formula and survival during subsequent storage

**DOI:** 10.3389/fmicb.2026.1939855

**Published:** 2026-08-27

**Authors:** Gurjot Kaur, Megan L. Fay, Raswanth M. Raju, Bhavya S. Pendyala, Shibali S. Alva, Robert Newkirk, Diana S. Stewart, Gregory Fleischman, Wei Zhang, Joelle K. Salazar

**Affiliations:** 1 Department of Food Science and Nutrition, Illinois Institute of Technology, Institute for Food Safety and Health, Bedford Park, IL, United States; 2 Division of Food Processing Science and Technology, U.S. Food and Drug Administration, Bedford Park, IL, United States

**Keywords:** *Cronobacter*, infant formula, predictive modeling, *Salmonella*, survival

## Abstract

Powdered infant formula (PIF) has been implicated as a potential vehicle for foodborne pathogens, such as *Cronobacter sakazakii* and *Salmonella enterica*, which can cause life-threatening infections in infants. For high-risk infants, public health agencies recommend using hot water to reconstitute PIF for safety. This study evaluated the influence of reconstitution water temperature on pathogen inactivation and subsequent survival and growth during storage. Milk-based (MB) and soy-based (SB) PIF were inoculated with *C. sakazakii* or *S. enterica* via spray atomization and left to stabilize for 1 month. Inoculated PIF was reconstituted using water at 25, 45, 70, 85, or 100 °C. A low-volume (LV) and a high-volume (HV) preparation were assessed. The dynamic log-linear model was applied to describe pathogen inactivation kinetics, and storage studies were subsequently conducted at 5, 10, and 25 °C for up to 48 h. Increasing the initial water temperature significantly enhanced pathogen inactivation in PIF, with substantial population reductions (>4 log CFU/mL) achieved with 85 and 100 °C water. Reconstitution of PIF at 70 °C provided moderate inactivation (2–3 log CFU/mL reduction). Sample volume had inconsistent effects on inactivation rates, with HV samples exhibiting slower cooling but not always greater pathogen reductions. Both pathogens demonstrated heat resistance, with observed outgrowth of *S. enterica* in SB-PIF at certain volume-temperature combinations. During storage of reconstituted PIF, both pathogens demonstrated regrowth at 25 °C, minimal to no growth at 10 °C, and no growth at 5 °C after 48 h. These findings highlight the need for immediate consumption or refrigeration of reconstituted PIF after preparation. The results of this study provide information on the safe preparation and handling of PIF by quantifying temperature-dependent pathogen inactivation in two different formulations.

## 1 Introduction

Powdered infant formula (PIF) has been identified as a potential vehicle for opportunistic foodborne pathogens, particularly *Cronobacter sakazakii* (formerly *Enterobacter sakazakii*) and *Salmonella enterica*. Infections caused by these pathogens can lead to severe outcomes, including meningitis, necrotizing enterocolitis, septicemia, and death, especially among neonates, infants born prematurely, and infants with weakened immune systems (Bowen and Braden, 2006; Strysko et al., 2020). Outbreaks linked to PIF have been reported globally, prompting increased attention from public health agencies and manufacturers (van Acker et al., 2001; Brouard et al., 2007; Cahill et al., 2008, Haston et al., 2023).

Contamination of PIF can occur through two primary routes: intrinsically during manufacturing and extrinsically after the container is opened. Intrinsic contamination has been documented in several outbreaks where *C. sakazakii* (*Enterobacter sakazakii*) was recovered from unopened, sealed containers (van Acker et al., 2001; CDC, 2002). Additional outbreaks with confirmed or suspected intrinsic contamination have been reported in France (Caubilla-Barron et al., 2007; Jourdan-da Silva et al., 2018). The addition of heat labile nutrients to PIF following the pasteurization step introduces the possibility of contamination, particularly for *C. sakazakii* and *S. enterica* which can persist in dry, low-moisture environments. Extrinsic contamination of PIF in home and healthcare settings appears to be more common (Strysko et al., 2020). Investigations have linked infant *C. sakazakii* infections to opened PIF containers, blenders used to mix PIF and water, and surfaces in the home kitchen environment (Block et al., 2002; Strysko et al., 2020; Haston et al., 2023). These findings highlight the ubiquitous nature of *C. sakazakii* in various environments and the ability of the pathogen to cross-contaminate PIF.

The possibility of intrinsic and extrinsic contamination of PIF underscores the importance of both manufacturing quality control and safe preparation practices by caregivers. Current recommendations from public health agencies emphasize the use of hot water to reconstitute PIF for high-risk infants; these recommendations generally include steps of boiling water, letting water cool for 5 min, then pouring the water into a bottle and mixing in PIF (CDC, 2024; FDA, 2024). The World Health Organization (WHO) recommends the use of water no < 70 °C for reconstitution of PIF, as water at this temperature dramatically reduces *C. sakazakii* populations and thus the risk to infants (WHO, 2007). However, studies have shown that caregivers and healthcare providers may deviate from these guidelines. For example, some caregivers may use cooler water to preserve perceived PIF quality, despite potential microbiological risks (Agostoni et al., 2004). Surveys of home and healthcare environments indicate that water temperatures during PIF reconstitution were frequently below 70 °C (Carletti and Cattaneo, 2008; Redmond and Griffith, 2013; Grant et al., 2024).

A recent study examining initial water temperatures for PIF reconstitution determined that *C. sakazakii* could be reduced by > 5 log CFU/mL with a water temperature of 73.8 °C when swirling the bottle and then allowing passive cooling to occur (Beary et al., 2025). The authors noted that passive cooling of the reconstituted PIF in the bottles at room temperature extended the thermal treatment of *C. sakazakii*, whereas active cooling, such as running cool water over the outside of the bottles after mixing, lead to limited thermal inactivation. Other studies have also determined that water temperatures ≥ 70 °C are capable of reducing *C. sakazakii* during PIF or powdered milk reconstitution, however, to varying degrees (Edelson-Mammel and Buchanan, 2004; Osaili et al., 2009a). One study reported that the use of 70 °C water resulted in a > 4 log CFU/mL reduction of *C. sakazakii* in reconstituted PIF (Edelson-Mammel et al., 2005). Studies evaluating water temperatures < 70 °C have observed minimal reductions of *C. sakazakii* during PIF reconstitution (Al-Holy et al., 2009; Garbaj et al., 2023).

Beyond the initial inactivation during reconstitution, the potential for pathogen growth during passive cooling and storage of reconstituted PIF may represent a food safety concern. Studies have shown that *C. sakazakii* can grow in reconstituted PIF held at ambient room temperature (Iversen et al., 2004; Parra-Flores et al., 2015). A growth kinetics study determined that even a low level of *C. sakazakii* (1.71 log CFU/mL) could reach high populations when reconstituted PIF was stored at room temperature for extended periods (Parra-Flores et al., 2015). Results of the study showed that *C. sakazakii* exhibited generation times of 0.67 and 0.41 h in reconstituted PIF at 22 and 35 °C, respectively.

While prior studies have determined that ≥ 70 °C reconstitution water temperature reduces *C. sakazakii* populations in PIF under some circumstances, this study extends that literature by aiming to (1) directly compare *C. sakazakii* and *S. enterica* under identical reconstitution conditions, (2) evaluate milk-based (MB) and soy-based (SB) PIF formulations and low-volume (LV) and high-volume (HV) preparations in parallel to determine whether these practical variables modify inactivation, (3) apply a dynamic log-linear kinetic model to characterize inactivation as a continuous function of temperature and time, and (4) link reconstitution temperature to subsequent regrowth risk during storage at refrigeration and abuse temperatures. Results from this study will aid in understanding the survival of *C. sakazakii* and *S. enterica* in reconstituted PIF.

## 2 Materials and methods

### 2.1 Strains and culture conditions

A four-strain cocktail of either *Cronobacter sakazakii* or *Salmonella enterica* was used in this study. For *C. sakazakii*, the strains included 600 (C713; powdered infant formula isolate), 589 (C702; food manufacturing environmental isolate), 587 (C704; food manufacturing environmental isolate), and 569 (FSM373). For *S. enterica*, the strains included Enteritidis PT30 (ATCC BAA-1045; almonds isolate), Agona [447967; roasted oats cereal isolate (CDC, 1998)], Alachua [CFSAN107331; peach leaf isolate (FDA, 2020)], and Poona [8785; CFSAN038692; cucumber isolate (CDC, 2016)]. These strains were selected based on their confirmed heat and desiccation tolerance. All *S. enterica* strains were rifampicin resistant (100 μg/mL). The rifampicin-resistance marker was verified not to alter thermal resistance or growth fitness relative to the wild-type strains. Each strain was cultured individually in Tryptic Soy Broth (TSB; Becton, Dickinson and Co., Sparks, MD, United States) for 16–18 h at 37 °C. Subsequently, 100 μL of each culture was individually plated onto Tryptic Soy Agar (TSA; Becton, Dickinson and Co.) and incubated at 37 °C for 24 h. Cells were harvested from agar plates using a sterile spreader with 1 mL Butterfields’s Phosphate Buffer (BPB, pH 7.2). Harvested cells were combined in equal volumes to create a four-strain cocktail of either *C. sakazakii* or *S. enterica* at a population level of ca. 10–11 log CFU/mL. To verify the initial population levels, both cocktails were serially diluted in BPB and plated onto Brain Heart Infusion Agar (BHIA; Becton, Dickinson and Co.). Agar plates were incubated at 37 °C for 24–48 h prior to enumeration.

### 2.2 Powdered infant formula (PIF) inoculation

A Milk-based (MB) and Soy-based (SB) powdered infant formula (PIF) from the same manufacturer were purchased from retail grocers in IL, United States. Both products are commercially available formulas labeled for infants 0–12 months of age. The main ingredients in the MB-PIF (comprising > 98%) were nonfat milk, lactose, vegetable oil (palm olein, coconut, soy, and high oleic sunflower oils), and whey protein concentrate. The main ingredients in the SB-PIF (comprising > 98%) were corn syrup solids, vegetable oil (palm olein, coconut, soy, and high oleic sunflower oils), and soy protein isolate. All PIF was stored in original containers (sealed cans of 354 g each) at room temperature (20–22 °C) and used within 1 month. Background microbiota in the uninoculated MB-PIF and SB-PIF was enumerated on BHIA at approximately 1 log CFU/g for both formulations.

Both MB-PIF and SB-PIF were artificially inoculated with the *C. sakazakii* or *S. enterica* cocktail at ca. 7–8 log CFU/g via spray atomization as previously described (Fay et al., 2025). Briefly, 1 kg of MB-PIF or SB-PIF was placed into the bowl of a stand mixer (N50A 1725 RPM 1/6 HP; Hobart Corporation, Troy, OH, United States) fixed with a paddle attachment. One of the inoculum cocktails (10 mL) was drawn into a syringe which was then screwed into an atomizing probe (Atomizing Horn 40 kHz 630-0499-07, Sonics & Materials Inc., Newtown, CT, United States). After starting the mixer at the lowest setting, the cocktail was sprayed via atomization over the PIF using an ultrasonic processor (VCX134PB 130W 40 kHz, Sonics & Materials Inc.) and converter (CV244 Sonics & Materials Inc.) for 1 min. The PIF was mixed on the lowest setting for 1 h, with bowl scrapes every 15 min. The 1 kg of inoculated PIF was then transferred into the metal bowl of a stand mixer (HL120 825 RPM 1/2 HP, Hobart Corporation) and combined with an additional 3 kg of uninoculated PIF. The 4 kg of PIF was mixed at the lowest setting for 1 h, with bowl scrapes every 15 min.

The procedure was repeated for each formulation so that each cocktail was independently inoculated. Inoculated MB-PIF and SB-PIF were stored at 25 °C and 33% relative humidity for 1 month to allow the pathogen populations to stabilize prior to use (Fay et al., 2025).

### 2.3 Powdered infant formula (PIF) reconstitution

Inoculated MB-PIF and SB-PIF were reconstituted using water at five different temperatures (25, 45, 70, 85, or 100 °C) to evaluate the effect of reconstitution temperature on pathogen inactivation. Two sample volumes were prepared in duplicates for each temperature for each trial: a low-volume (LV) consisting of 8.8 g PIF (1 scoop) reconstituted with 59.2 mL water and a high-volume (HV) consisting of 35.2 g PIF (4 scoops) reconstituted with 236.8 mL water. The LV and HV reconstitutions were prepared in 125 and 500-mL polypropylene plastic bottles, respectively. These LV and HV reconstitutions corresponded to the lowest and highest volumes (fl. oz. bottle) which could be prepared according to the manufacturer’s instructions provided on the PIF product label on the container.

Autoclaved tap water was heated to the target temperature of 25, 45, 70, 85, or 100 °C ( ± 0.1 °C) and measured using a digital data logging thermometer with a thermocouple (Fluke 52 II B; Fluke Corporation, Everett, WA, United States). Water was added to the bottles containing the weighed PIF, and bottles were immediately capped and swirled by hand for 5 s to ensure uniform mixing. The reconstituted PIF was left on the counter at ambient temperature (20–22 °C), allowing natural cooling to occur. The temperature of the reconstituted PIF was recorded immediately after mixing (time 0) and at intervals throughout 240 min (4 h).

Samples were collected for pathogen enumeration at 0, 1, 3, 5, 10, 30, 60, 120, and 240 min. At each sampling time, bottles were mixed by inversion before removing a 1-mL aliquot for serial dilution and enumeration (see section 2.5). For trials with initial water temperatures of 70, 85, and 100 °C, sample aliquots were removed and placed immediately on ice. Three independent trials were conducted for each combination of water temperature, sample volume, PIF formulation, and pathogen (*n* = 6).

### 2.4 Storage of reconstituted powdered infant formula (PIF)

Following the 240-min (4 h) cooling of reconstituted PIF, 4 mL from each duplicate bottle was transferred into 15-mL tubes for storage. Tubes were loosely capped and placed at 5, 10, or 25 °C for up to 48 h. Sampling was performed after 24 and 48 h by removing a 1-mL aliquot for serial dilution and enumeration (see section 2.5).

### 2.5 Pathogen enumeration from reconstituted powdered infant formula (PIF)

*C. sakazakii* and *S. enterica* samples were serially diluted in Butterfield’s Phosphate Buffer (BPB, pH 7.2). *C. sakazakii* sample dilutions were plated onto BHIA, while *S. enterica* sample dilutions were plated onto BHIA supplemented with rifampicin (100 μg/mL). All agar plates were incubated at 37 °C for 24–48 h before enumeration. *C. sakazakii* colonies were identified based on characteristic colony morphology on BHIA. Suspect or atypical colonies were confirmed using Bio-Rad RAPID’*Sakazakii* agar (Bio-Rad Laboratories, Hercules, CA, United States). *C. sakazakii* and *S. enterica* populations were expressed as log CFU/mL. The limit of enumeration of the plate count assay was 1.39 log CFU/mL.

### 2.6 Modeling of pathogen inactivation kinetics

The inactivation of *C. sakazakii* and *S. enterica* in reconstituted PIF was modeled using a dynamic log-linear approach as described previously (Salazar et al., 2024). Briefly, the temperature data during cooling for each trial was modeled, which indicated first order cooling, as in Equation 1.


$$l\,n\,\frac{T-T_{f}}{T_{0}-T_{f}}=-\frac{t}{\tau }$$ (1)

where *T*_0_ is the zero-time temperature, *T*_*f*_ is the final temperature, and τ is the time constant.

The isothermal log-linear model (Bigelow and Esty, 1920) was modified to incorporate temperature changes as the reconstituted PIF cooled. A dynamic log-linear model was fit to pathogen populations (log CFU/mL) using the Solver add-in in Microsoft Excel. Since inactivation occurred during cooling, the model incorporated temperature changes over time. The inactivation is given by Equation 2:


$$l\,o\,g\,\frac{N_{0}}{N}=\frac{1}{D_{T_{r\,e\,f}}}\,\int_{0}^{t}10^{\left(T\,\left(\theta \right)-T_{r\,e\,f}\right)/z}\,d\theta$$ (2)

Where *N* is the population at time *t*, *N*_0_ is the initial population, *D*_*T_ref_*_ is the decimal reduction time at temperature *T*_*ref*_ (set at a reference temperature of 70 °C), *T* is the temperature at which inactivation occurs, *z* is the *z* value, θ is the integration variable representing time, and *T(θ)* is the variation in temperature over the inactivation time.

Resulting *D* and *z* values were converted to inactivation rates (*k*_*max*;_ log CFU/mL permin) as in Equation 3:


$$k_{m\,a\,x}=\frac{l\,n\,10}{D_{T_{r\,e\,f}}}\,10^{\frac{T_{0}-T_{r\,e\,f}}{z}}$$ (3)

Model fits were evaluated by minimizing the sum of squared differences between calculated and observed reductions. Root mean square error (RMSE) was calculated for each trial. The number of time points contributing to the sum-of-squares minimization varied by trial: points were included up to and including the first observation at or below the limit of enumeration, after which subsequent censored (non-quantifiable) observations were excluded from the fit. Depending on the rate of inactivation, this resulted in 2–7 points entering the fit for a given trial.

### 2.7 Secondary modeling

Secondary modeling was conducted using the Ratkowsky square root model (Ratkowsky et al., 1982) to ascertain the relationship between pathogen inactivation rate (*k*_*max*_) and reconstitution water temperature. The *k*_*max*_ values from individual trials were plotted against the zero-time water temperature (*T*_0_) for each pathogen, reconstitution sample volume, and PIF type combination. Linear regression was used to assess these relationships, and the goodness of fit was examined using the coefficient of determination (*r*^2^). The regression slope with its 95% confidence interval (CI) was reported.

### 2.8 Statistical analysis

Three independent trials were performed for each combination of PIF formulation, initial water temperature (*T*_*i*_), pathogen, and sample volume. Differences in pathogen populations in PIF during reconstitution or storage were statistically analyzed using ANOVA with Tukey’s *post-hoc* test (α = 0.05). Populations below the limit of enumeration (1.39 log CFU/mL) were recorded as 1.39 log CFU/mL for the purposes of statistical analyses and log reduction calculations. An inactivation rate (*k*_*max*_) was obtained for each replicate for each independent trial (*n* = 6); root mean square error (RMSE) was reported for each *k*_*max*_ value. Differences in mean *k*_*max*_ values between different initial water temperatures (*T*_*i*_) were statistically analyzed using ANCOVA with Tukey’s *post hoc* test (α = 0.05). Prior to analysis, the assumptions of normality of residuals and homogeneity of variance were assessed using the Shapiro-Wilk and Levene tests, respectively. Because each of the three independent trials included two duplicate bottles, the duplicate measurements were treated as technical replicates nested within trial rather than as independent observations.

## 3 Results

### 3.1 Temperature dynamics of reconstituted powdered infant formula (PIF)

During reconstitution of PIF at different initial water temperatures (*T*_*i*_ = 25, 45, 70, 85, or 100 °C), temperatures decreased over the 240 min (4 h) period due to natural cooling at ambient laboratory temperature (20–22 °C) (Supplementary Figure 1). Supplementary Figure 1 displays the natural cooling temperature profiles of the reconstituted PIF over the first 60 min period. The rate of cooling depended on both the initial water temperature (*T*_*i*_) and the sample volume (LV or HV). No significant differences in cooling were observed between the two PIF formulations (MB or SB). Overall, the zero-time temperature (*T*_0_) after mixing the PIF and water was up to 20 °C lower than the *T*_*i*_.

For samples reconstituted at 100 °C, the zero-time temperature (*T*_0_) after mixing the PIF and water ranged from 77.1 to 87.5 °C for LV and 83.1–88.5 °C for HV samples, temperature decreases of up to 20 °C from the initial water temperature (*T*_*i*_). Similar initial temperature reductions were observed for the other reconstituted samples in this study. For samples reconstituted at 85 °C, the zero-time formula temperature (*T*_0_) after mixing ranged from 68.3 to 78.8 °C for LV and 74.3–80.2 °C for HV samples, temperature decreases of 4.8–16.7 °C from the initial water temperature (*T*_*i*_). For samples reconstituted at 70 °C, the zero-time formula temperature (*T*_0_) after mixing ranged from 52.0 to 63.4 °C for LV and 61.0–68.9 °C for HV samples, temperature decreases of 1.1–18.0 °C from the initial water temperature (*T*_*i*_). Less dramatic temperature decreases were observed for the samples reconstituted with water at the lower temperatures. For the samples reconstituted at 45 °C, only minimal temperature decreases of 2.0–4.4 °C for LV and 0.2–1.9 °C for HV samples were observed. For samples reconstituted at 25 °C, no significant initial decrease in temperature was observed for either LV or HV samples.

By 240 min (4 h), temperatures of all samples decreased to 21–27 °C, with the higher temperatures resulting from the use of 100 °C water (*T*_*i*_) with the HV samples. Overall, HV samples exhibited slower cooling rates and maintained elevated temperatures longer than LV samples at the same initial water temperature (*T*_*i*_).

### 3.2 Inactivation of *Cronobacter sakazakii* in reconstituted powdered infant formula (PIF)

**FIGURE 1:**
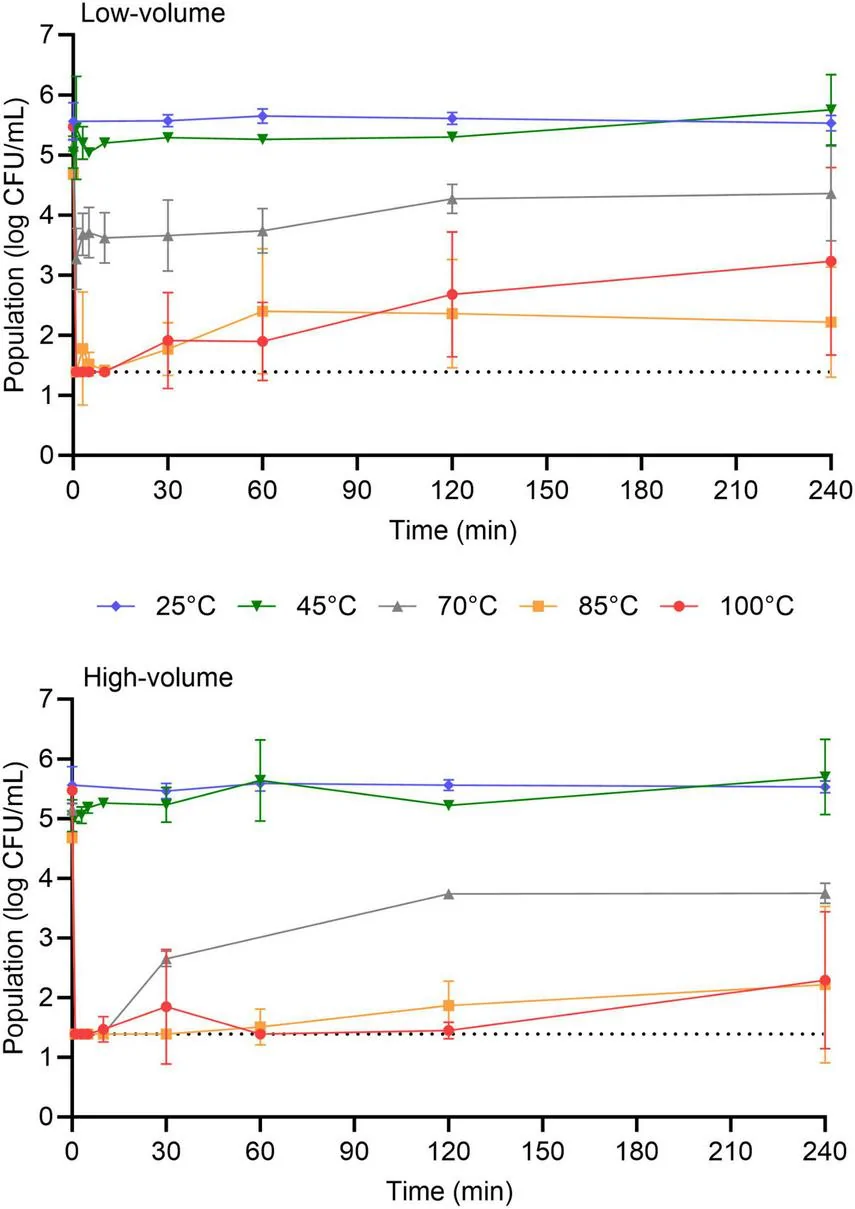
Populations of *Cronobacter sakazakii* in a low-volume (LV) or high-volume (HV) of milk-based (MB) powdered infant formula (PIF) reconstituted with an initial water temperature (*T*_*i*_) of 25, 45, 70, 85, or 100 °C and left on the counter at room temperature (20–22 °C) for up to 240 min. Data are means ± standard deviations (*n* = 6). Dotted line indicates the lower limit of enumeration (1.39 log CFU/mL).

**FIGURE 2:**
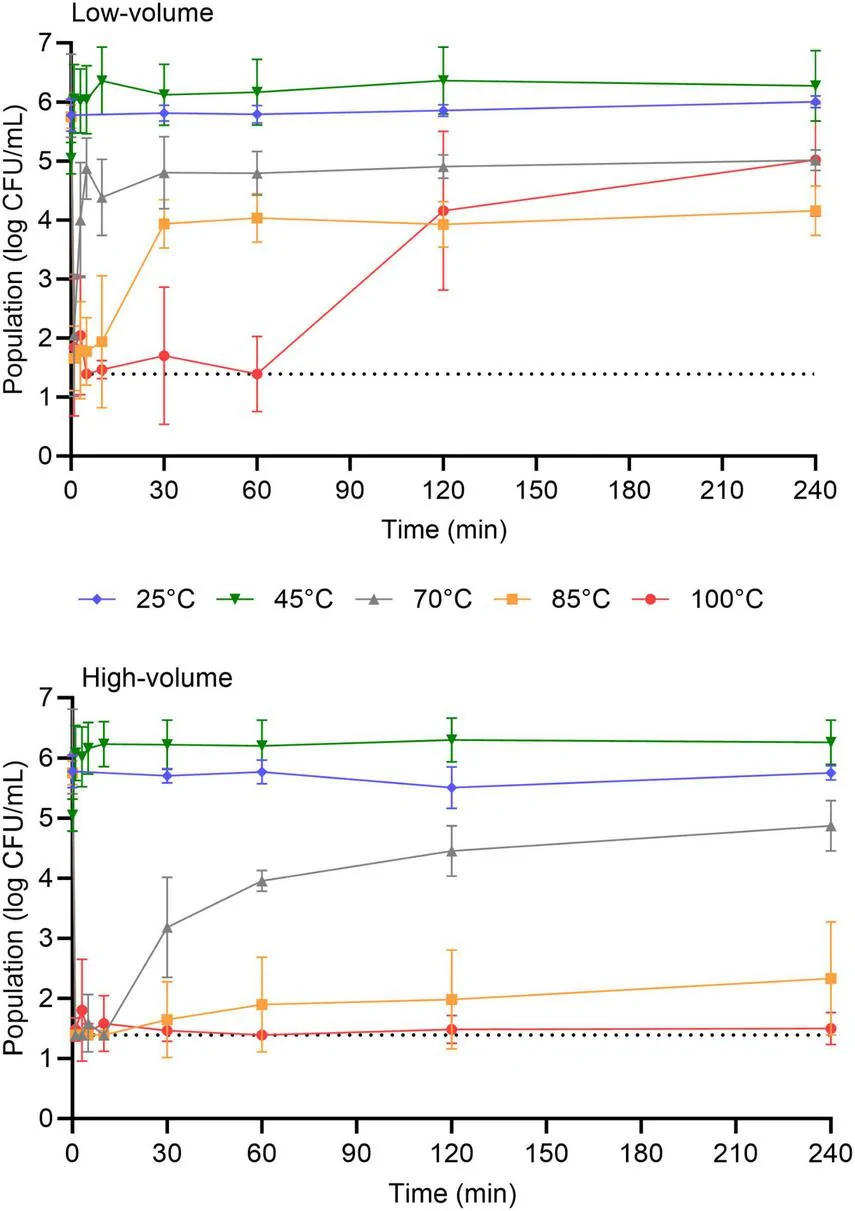
Populations of *Cronobacter sakazakii* in a low-volume (LV) or high-volume (HV) of soy-based (SB) powdered infant formula (PIF) reconstituted with an initial water temperature (*T*_*i*_) of 25, 45, 70, 85, or 100 °C and left on the counter at room temperature (20–22 °C) for up to 240 min. Data are means ± standard deviations (*n* = 6). Dotted line indicates the lower limit of enumeration (1.39 log CFU/mL).

Reconstitution temperature had a pronounced effect on *C. sakazakii* populations in PIF (Figures 1, 2). Overall, the initial levels of inactivation (within the first 5 min) depended on the initial (*T*_*i*_) and zero-time (*T*_0_) water temperatures. When water at 25 and 45 °C *T*_*i*_ was used for reconstitution, no significant inactivation occurred and *C. sakazakii* populations remained stable throughout the 240 min (4 h) period. At 70 °C initial water temperature (*T*_*i*_), *C. sakazakii* populations decreased in the LV samples by 1.89 and 4.06 log CFU/mL within the first 5 min for MB- and SB-PIF, respectively, with no further change during the remaining period in the MB-PIF. *C. sakazakii* increased in population by approximately 2.5 log CFU/mL in the SB-PIF after 10 min, then the population remained stable for the remaining period. For HV samples reconstituted at 70 °C, regardless of formulation, *C. sakazakii* was initially reduced to < 1.39 log CFU/mL. However, populations subsequently increased to 2.65 and 3.18 log CFU/mL after 30 min in the MB- and SB-PIF, respectively; populations of 3.74 and 4.45 log CFU/mL were subsequently achieved after 120 min, respectively (overall increases of approximately 2.5 and 3 log CFU/mL, respectively).

Reconstitution at 85 °C initial water temperature (*T*_*i*_) resulted in initial population reductions to 1.66 log CFU/mL (for SB-PIF LV samples) and < 1.39 log CFU/mL for all other samples. *C. sakazakii* in the HV samples showed minimal or no increase in population during passive cooling, whereas the pathogen in the LV samples significantly increased. The *C. sakazakii* population in the MB-PIF LV samples increased by approximately 1 log CFU/mL after 240 min (4 h), whereas the population in the SB-PIF samples increased by approximately 3 log CFU/mL after 30 min with no further significant change for the remaining period.

Reconstitution at 100 °C initial water temperature (*T*_*i*_) resulted in initial population reductions to < 1.39 log CFU/mL for the MB-PIF samples, regardless of sample volume; population reductions to 1.84 and 1.48 log CFU/mL were achieved for the SB-PIF LV and HV samples, respectively. No significant population change was observed in the MB-PIF HV samples; however, an increase of approximately 2 log CFU/mL was observed for the LV samples after 240 min (4 h). Populations of *C. sakazakii* in the SB-PIF LV samples increased by approximately 3 log CFU/mL after 120 min and by 4 log CFU/mL after 240 min (4 h). No significant increase in population was observed in the SB-PIF HV samples.

### 3.3 Inactivation of *Salmonella enterica* in reconstituted powdered infant formulas

**FIGURE 3:**
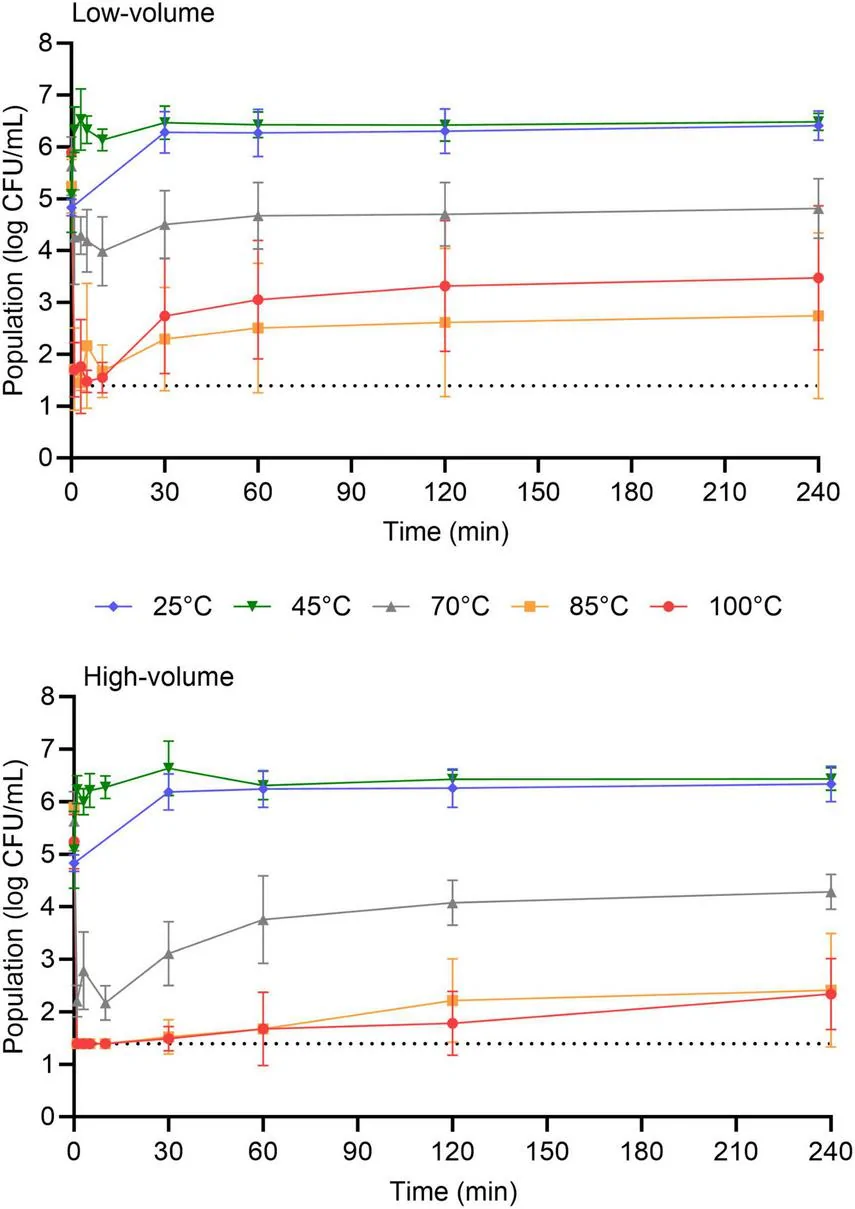
Populations of *Salmonella enterica* in a low-volume (LV) or high-volume (HV) of milk-based (MB) powdered infant formula (PIF) reconstituted with an initial water temperature (*T*_*i*_) of 25, 45, 70, 85, or 100 °C and left on the counter at room temperature (20–22 °C) for up to 240 min. Data are means ± standard deviations (*n* = 6). Dotted line indicates the lower limit of enumeration (1.39 log CFU/mL).

**FIGURE 4:**
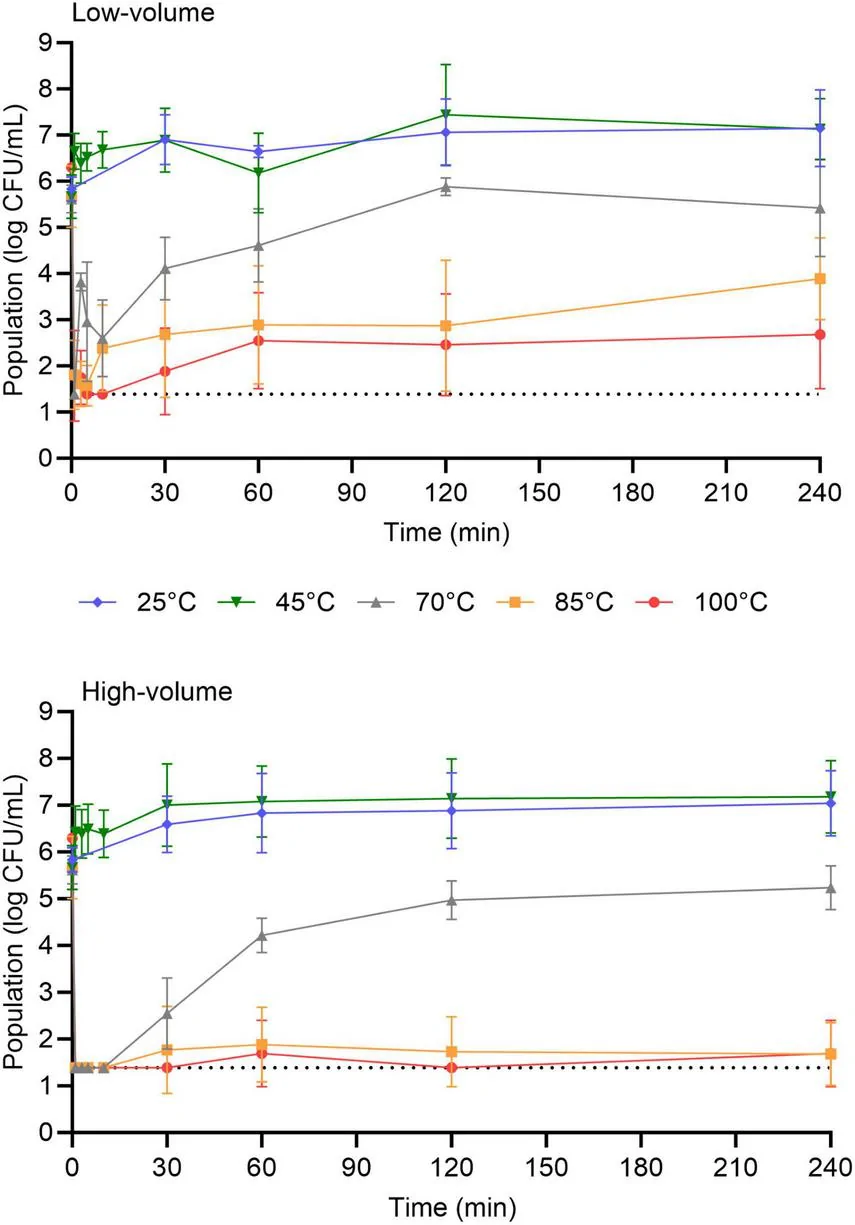
Populations of *Salmonella enterica* in a low-volume (LV) or high-volume (HV) of soy-based (SB) powdered infant formula (PIF) reconstituted with an initial water temperature (*T*_*i*_) of 25, 45, 70, 85, or 100 °C and left on the counter at room temperature (20–22 °C) for up to 240 min. Data are means ± standard deviations (*n* = 6). Dotted line indicates the lower limit of enumeration (1.39 log CFU/mL).

*S. enterica* demonstrated a similar inactivation response as *C. sakazakii* in MB- and SB- PIF (Figures 3, 4). As with *C. sakazakii*, populations of *S. enterica* in PIF reconstituted at 25 and 45 °C initial water temperature (*T*_*i*_) were not inactivated and remained stable throughout the 240 min (4 h) period. When PIF was reconstituted with 70 °C initial water temperature (*T*_*i*_), *S. enterica* populations in the HV samples were initially inactivated (within the first 5 min) to a greater degree than in the LV samples, regardless of PIF type. Specifically, populations in MB-PIF were initially reduced by 1.37 and 3.42 log CFU/mL in the LV and HV samples, respectively. Populations in the SB-PIF were initially reduced by ≥ 4.23 log CFU/mL in both sample volumes. Following initial inactivation, the populations in the MB-PIF significantly increased by approximately 1 and 2 log CFU/mL in the LV and HV samples, respectively. The SB-PIF allowed for more rapid recovery to higher populations during passive cooling with > 3 log CFU/mL increases in *S. enterica* populations after only 120 min in both sample volumes.

Reconstitution at 85 °C initial water temperature (*T*_*i*_) achieved initial *S. enterica* population reductions of 3.53 and ≥ 3.85 log CFU/mL in the LV and HV samples, respectively, in the MB-PIF. Populations in the SB-PIF were initially reduced by 3.86 and ≥ 4.28 log CFU/mL in the LV and HV samples, respectively. While no significant increase in population was observed in the SB-PIF HV samples during the remaining 240 min (4 h) cooling period, an increase of approximately 1 log CFU/mL occurred in the MB-PIF HV samples. In the LV samples, population increases of approximately 1 and 2 log CFU/mL occurred in the MB- and SB-PIF samples, respectively.

Reconstitution at 100 °C initial water temperature (*T*_*i*_) resulted in initial *S. enterica* population reductions to < 1.39 log CFU/mL for the HV samples, regardless of PIF type. Initial population reductions to 1.70 and 1.79 log CFU/mL were achieved in the MB- and SB-PIF LV samples, respectively. Similar to the 85 °C initial water temperature (*T*_*i*_), no significant increase in *S. enterica* population was observed in the SB-PIF HV samples during the remaining 240 min (4 h) cooling period, while an increase of approximately 1 log CFU/mL occurred in the MB-PIF HV samples. In the LV samples, population increases of approximately 1.5 and 1 log CFU/mL occurred in the MB- and SB-PIF samples, respectively, after only 60 min of cooling.

### 3.4 Primary modeling of pathogen inactivation kinetics

**FIGURE 5:**
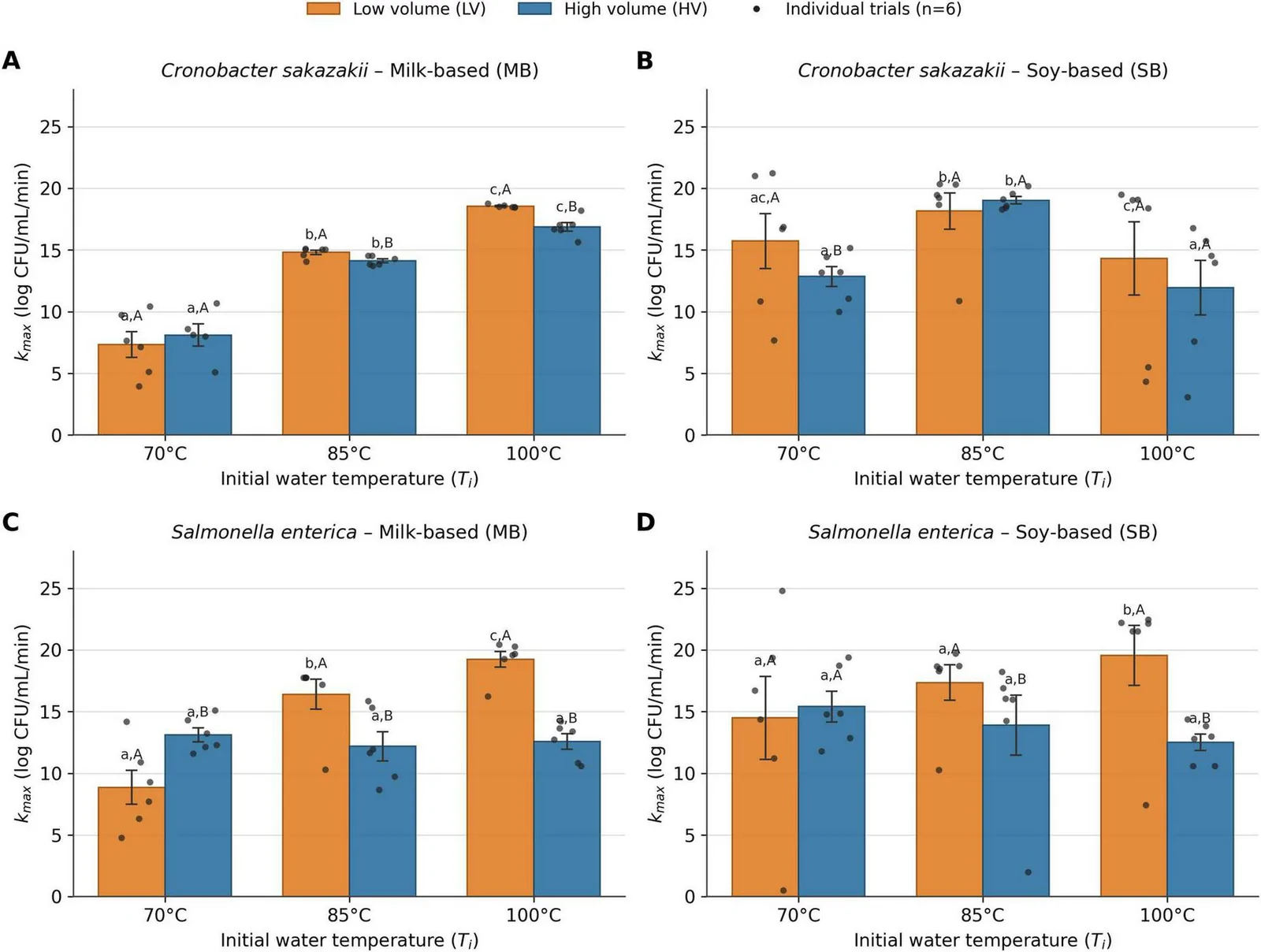
Inactivation rates (*k*_max_; log CFU/mL per min) of *Cronobacter sakazakii* in reconstituted **(A)** milk-based (MB) and **(B)** soy-based (SB) powdered infant formula (PIF) and *Salmonella enterica* in reconstituted **(C)** milk-based (MB) and **(D)** soy-based (SB) PIF based on initial (*T*_*i*_) water temperatures of 70, 85, or 100 °C. Mean *k*_max_ values with different lowercase letters are significantly different for the same volume between different *T*_*i*_. Mean *k*_max_ values with different uppercase letters are significantly different between different volumes at the same *T*_*i*_.

Kinetic parameters of the dynamic log-linear model describing the inactivation for *C. sakazakii* in reconstituted MB- and SB-PIF are displayed in Tables 1, 2 and Figures 5A,B, respectively, whereas the parameters for *S. enterica* are shown in Tables 3, 4 and Figures 5C,D. For *C. sakazakii* in MB-PIF, maximum inactivation rates (*k*_*max*;_ log CFU/mL per min) ranged from an average of 6.76 when 70 °C initial water temperature was used to an average of 18.55 log CFU/mL per min when 100 °C initial water temperature was used. *C. sakazakii* in HV samples exhibited similar but slightly lower *k*_*max*_ values at equivalent temperatures. Mean *k*_*max*_ values in SB-PIF for LV and HV samples were 15.73 and 12.85 log CFU/mL per min at 70 °C, 18.16 and 19.03 log CFU/mL per min at 85 °C, and 14.31 and 11.95 log CFU/mL per min at 100 °C, respectively. Unlike the results for MB-PIF, inactivation of *C. sakazakii* in SB-PIF was not well correlated with sample volume or reconstitution water temperature.

**TABLE 1:** Kinetic parameters of the dynamic log-linear model to describe the inactivation of *Cronobacter sakazakii* in reconstituted milk-based (MB) powdered infant formula (PIF) based on initial (*T*_*i*_) water temperatures of 70, 85, or 100 °C.

*T*_*i*_ (°C)^a^	Volume^b^	*T*_0_ (°C)^c^	*k*_*max*_^d^ (log CFU/mL per min)	RMSE^e^	Mean *k*_*max*_^f^ (log CFU/mL per min ± SE^g^)
70	Low	58.8	5.12	0.1193	7.34 ± 0.42 aA
		59.2	3.95	0.0528	
		60.0	10.42	0.1081	
		60.9	9.75	0.0773	
		61.2	7.66	0.2323	
		62.1	7.14	0.2319	
	High	62.7	5.10	0.1713	6.76 ± 0.63 aA
		63.6	N/A^h^	N/A	
		64.5	8.01	0.2220	
		64.8	10.70	0.0753	
		64.9	8.60	0.1259	
		65.8	8.12	0.1155	
85	Low	70.1	15.11	0.1668	14.81 ± 0.07 bA
		70.7	15.03	0.0028	
		72.7	14.59	0.1668	
		74.7	15.03	0.2018	
		78.3	14.05	0.4723	
		78.8	15.04	0.1813	
	High	74.3	14.54	0.0136	14.13 ± 0.06 bB
		74.6	14.53	0.0136	
		78.4	13.72	0.0336	
		78.6	13.85	0.0300	
		78.8	14.27	0.1114	
		78.9	13.85	0.0253	
100	Low	80.1	18.48	0.0069	18.55 ± 0.02 cA
		80.2	18.49	0.0066	
		80.3	18.45	0.0581	
		81.2	18.52	0.0061	
		84.0	18.77	0.2973	
		85.0	18.61	0.2895	
	High	83.1	16.62	0.0456	16.88 ± 0.14 cB
		83.2	16.69	0.0448	
		86.9	17.06	0.0372	
		87.0	15.64	0.1018	
		88.0	18.21	0.3458	
		88.2	17.04	0.0379	

^a^, *T*_*i*_, initial temperature of the water;

^b^, volume, low volume was 1 scoop (8.8 g) of powdered infant formula and 59.2 mL of water while high volume was 4 scoops (35.2 g) and 236.8 mL of water;

^c^, *T*_0_, temperature of the reconstituted powdered infant formula immediately after water addition;

^d^, *k*_max_, inactivation rate (log CFU/mL per min), each value represents an individual trial;

^e^, root mean square error;

^f^, mean *k*_max_ from 6 individual trials at the same *T*_*i*_;

^g^, SE, standard error;

^h^, not applicable. Mean *k*_max_ values with different lowercase letters are significantly different for the same volume between different *T*_*i*_. Mean *k*_max_ values with different uppercase letters are significantly different between different volumes at the same *T*_*i*_.

**TABLE 2:** Kinetic parameters of the dynamic log-linear model to describe the inactivation of *Cronobacter sakazakii* in reconstituted soy-based (SB) powdered infant formula (PIF) based on initial (*T*_*i*_) water temperatures of 70, 85, or 100 °C.

*T*_*i*_ (°C)^a^	Volume^b^	*T*_0_ (°C)^c^	*k*_*max*_^d^ (log CFU/mL per min)	RMSE^e^	Mean *k*_*max*_^f^ (log CFU/mL per min ± SE^g^)
70	Low	58.7	16.73	1.5820	15.73 ± 0.91 acA
		59.2	21.22	1.6766	
		60.1	16.89	1.5398	
		60.1	21.00	1.7733	
		61.0	10.85	0.7370	
		62.3	7.69	0.3656	
	High	63.8	11.06	0.7402	12.85 ± 0.33 aB
		64.2	10.00	0.2251	
		64.8	15.18	0.5836	
		64.8	13.18	0.6384	
		65.6	14.45	0.4773	
		65.7	13.22	0.7185	
85	Low	69.9	18.69	0.4738	18.16 ± 0.60 bA
		70.1	19.25	0.4253	
		73.2	19.46	0.8041	
		74.0	10.87	0.4672	
		75.0	20.34	0.5449	
		75.2	20.33	0.3257	
	High	75.1	18.30	0.0212	19.03 ± 0.12 bA
		75.4	18.41	0.0188	
		79.3	18.59	0.0191	
		79.4	19.13	0.3155	
		79.6	20.20	0.2205	
		79.7	19.57	0.0336	
100	Low	80.9	4.34	0.5865	14.31 ± 1.21 cA
		81.1	5.52	0.2689	
		81.2	18.38	0.0698	
		81.8	19.05	0.0975	
		84.0	19.49	0.0107	
		86.5	19.07	0.0196	
	High	85.1	7.59	1.1827	11.95 ± 0.90 aA
		85.5	3.06	0.5668	
		86.1	15.74	0.2485	
		86.3	14.53	0.1174	
		86.5	13.97	0.1281	
		88.5	16.79	0.0688	

^a^, *T*_*i*_, initial temperature of the water;

^b^, volume, low volume was 1 scoop (8.8 g) of powdered infant formula and 59.2 mL of water while high volume was 4 scoops (35.2 g) and 236.8 mL of water;

^c^, *T*_0_, temperature of the reconstituted powdered infant formula immediately after water addition;

^d^, *k*_max_, inactivation rate (log CFU/mL per min), each value represents an individual trial;

^e^, root mean square error;

^f^, mean *k*_max_ from 6 individual trials at the same *T*_*i*_;

^g^, SE, standard error. Mean *k*_max_ values with different lowercase letters are significantly different for the same volume between different *T*_*i*_. Mean *k*_max_ values with different uppercase letters are significantly different between different volumes at the same *T*_*i*_.

**TABLE 3:** Kinetic parameters of the dynamic log-linear model to describe the inactivation of *Salmonella enterica* in reconstituted milk-based (MB) powdered infant formula (PIF) based on initial (*T*_*i*_) water temperatures of 70, 85, or 100 °C.

*T*_*i*_ (°C)^a^	Volume^b^	*T*_0_ (°C)^c^	*k*_*max*_^d^ (log CFU/mL per min)	RMSE^e^	Mean *k*_*max*_^f^ (log CFU/mL per min ± SE^g^)
70	Low	59.9	7.71	0.0144	8.86 ± 0.56 aA
		60.6	6.31	0.0051	
		61.9	9.29	0.3693	
		62.0	4.77	0.2514	
		62.2	14.19	0.4881	
		62.9	10.89	0.2731	
	High	63.5	15.09	0.0464	13.11 ± 0.23 aB
		63.8	12.14	0.1715	
		65.3	12.29	0.0561	
		65.0	14.32	0.1090	
		68.3	11.59	0.0706	
		68.9	13.25	0.0355	
85	Low	71.0	17.74	0.0000	16.41 ± 0.50 bA
		71.6	17.74	0.0000	
		72.0	17.74	0.0000	
		72.5	10.30	0.2268	
		72.6	17.74	0.0000	
		72.8	17.19	0.0580	
	High	74.8	15.85	0.0400	12.20 ± 0.48 aB
		75.0	15.31	0.0926	
		78.1	11.94	0.1236	
		79.1	11.66	0.1301	
		79.4	9.73	0.1751	
		79.6	8.67	0.2027	
100	Low	77.1	19.57	0.1606	19.25 ± 0.26 cA
		77.2	19.67	0.0685	
		80.4	20.29	0.0095	
		81.4	20.43	0.0064	
		81.4	16.23	0.2432	
		82.9	19.28	0.0444	
	High	85.1	14.23	0.1386	12.57 ± 0.25 aB
		85.2	12.72	0.1734	
		86.5	13.39	0.1574	
		86.6	10.84	0.2188	
		86.7	10.58	0.2255	
		87.0	13.66	0.1518	

^a^, *T*_*i*_, initial temperature of the water;

^b^, volume, low volume was 1 scoop (8.8 g) of powdered infant formula and 59.2 mL of water while high volume was 4 scoops (35.2 g) and 236.8 mL of water;

^c^, *T*_0_, temperature of the reconstituted powdered infant formula immediately after water addition;

^d^, *k*_max_, inactivation rate (log CFU/mL per min), each value represents an individual trial;

^e^, root mean square error;

^f^, mean *k*_max_ from 6 individual trials at the same *T*_*i*_;

^g^, SE, standard error. Mean *k*_max_ values with different lowercase letters are significantly different for the same volume between different *T*_*i*_. Mean *k*_max_ values with different uppercase letters are significantly different between different volumes at the same *T*_*i*_.

**TABLE 4:** Kinetic parameters of the dynamic log-linear model to describe the inactivation of *Salmonella enterica* in reconstituted soy-based (SB) powdered infant formula (PIF) based on initial (*T*_*i*_) water temperatures of 70, 85, or 100 °C.

*T*_*i*_ (°C)^a^	Volume^b^	*T*_0_ (°C)^c^	*k*_*max*_^d^ (log CFU/mL per min)	RMSE^e^	Mean *k*_*max*_^f^ (log CFU/mL per min ± SE^g^)
70	Low	52.0	24.79	0.1811	14.49 ± 1.38 aA
		52.0	19.36	0.0021	
		61.2	0.50	0.0779	
		62.0	16.71	0.0587	
		63.0	14.36	0.0524	
		63.4	11.22	0.1587	
	High	61.0	19.40	0.0012	15.40 ± 0.51 aA
		62.0	18.74	0.2250	
		63.6	12.87	0.1092	
		65.0	11.78	0.0653	
		65.4	14.77	0.0111	
		65.8	14.85	0.0000	
85	Low	68.3	10.28	0.2713	17.36 ± 0.58 aA
		68.4	18.29	0.1411	
		71.3	18.68	0.0550	
		71.3	18.71	0.0226	
		72.0	18.48	0.0932	
		72.2	19.71	0.0000	
	High	76.3	18.22	0.1713	13.91 ± 1.00 aB
		75.9	16.06	0.0774	
		79.1	14.26	0.1160	
		79.2	16.91	0.0076	
		79.9	2.00	0.0598	
		80.2	16.00	0.0188	
100	Low	77.7	7.42	0.0418	19.55 ± 0.99 bA
		77.9	22.46	0.0038	
		80.7	22.15	0.0102	
		82.5	21.51	0.0239	
		82.5	22.21	0.0089	
		87.5	21.51	0.0236	
	High	84.2	12.78	0.3230	12.52 ± 0.27 aB
		86.5	14.36	0.1781	
		86.5	13.85	0.1904	
		87.0	12.97	0.2105	
		88.2	10.58	0.2726	
		88.5	10.58	0.2724	

^a^, *T*_*i*_, initial temperature of the water;

^b^, volume, low volume was 1 scoop (8.8 g) of powdered infant formula and 59.2 mL of water while high volume was 4 scoops (35.2 g) and 236.8 mL of water;

^c^, *T*_0_, temperature of the reconstituted powdered infant formula immediately after water addition;

^d^, *k*_max_, inactivation rate (log CFU/mL per min), each value represents an individual trial;

^e^, root mean square error;

^f^, mean *k*_max_ from 6 individual trials at the same *T*_*i*_;

^g^, SE, standard error. Mean *k*_max_ values with different lowercase letters are significantly different for the same volume between different *T*_*i*_. Mean *k*_max_ values with different uppercase letters are significantly different between different volumes at the same *T*_*i*_.

Mean *k*_*max*_ values for *S. enterica* in MB-PIF LV samples were statistically different over the three initial water temperatures: 8.86, 16.41, and 19.25 log CFU/mL per min at 70, 85, and 100 °C, respectively. However, the mean *S. enterica k*_*max*_ values in the HV samples prepared with water at the same temperatures were not significantly different at 13.11, 12.20, and 12.57 log CFU/mL per min, respectively. *S. enterica k*_*max*_ values in SB-PIF were similar to those of MB-PIF. The mean *k*_*max*_ values in the SB-PIF LV samples were 14.49, 17.36, and 19.55 log CFU/mL per min when water at initial temperatures of 70, 85, and 100 °C was used, respectively. The mean *k*_*max*_ values in the HV samples were inversely correlated in the SB-PIF, with values decreasing from 15.40 at 70 °C, 13.91 at 85 °C and 12.52 at 100 °C. Overall, inactivation of *S. enterica* was not well correlated with sample volume or reconstitution water temperature.

### 3.5 Secondary modeling of *C. sakazakii* inactivation in reconstituted milk-based (MB) PIF

**FIGURE 6:**
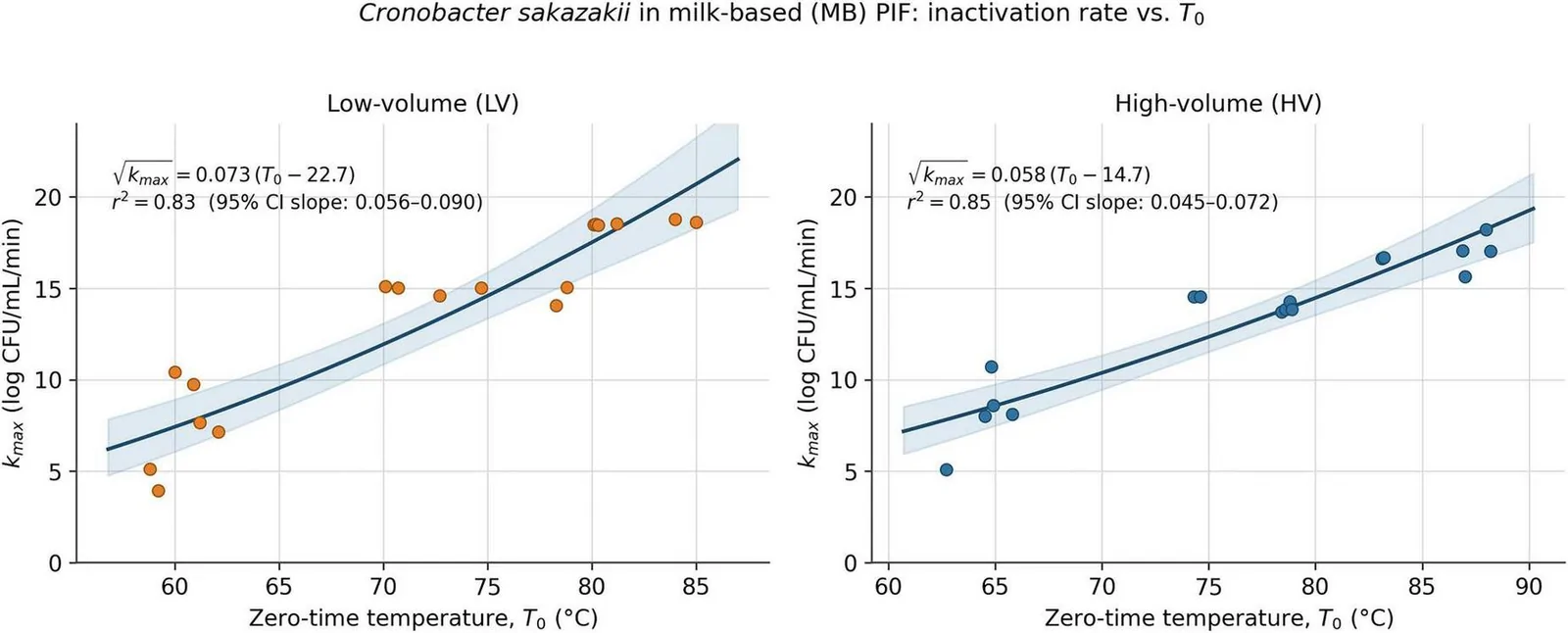
Relationship between the inactivation rate (*k*_max_; log CFU/mL per min) of *Cronobacter sakazakii* in a low-volume (LV) or high-volume (HV) of milk-based (MB) powdered infant formula (PIF) reconstituted with an initial water temperature (*T*_*i*_) of 70, 85, or 100 °C. Inactivation rates (*k*_max_) of *C. sakazakii* are shown *versus* time-zero temperature of water (*T*_0_). Each data point represents an individual trial for each of the three *T*_*i*_ (*n* = 18).

A strong linear relationship was observed between the maximum inactivation rate (*k*_*max*_) and the zero-time water temperature (*T*_0_; 70, 85, and 100 °C) for *C. sakazakii* in MB-PIF (Figure 6) with *r*^2^ values of 0.83 and 0.85 for LV and HV samples, respectively. The equation for *C. sakazakii* in LV samples was √*k*_*max*_ = 0.073*(*T*_0_–22.7) with satisfactory goodness-of-fit parameters (*r*^2^ = 0.83; 95% CI slope = 0.056–0.090). The equation for *C. sakazakii* in the HV samples was √*k*_*max*_ = 0.058*(*T*_0_–14.7), also with satisfactory goodness-of-fit parameters (*r*^2^ = 0.85; 95% CI slope-0.045–0.072). The limited number of temperature levels (70, 85, and 100 °C) constrains extrapolation beyond the 70–100 °C range tested in this study. Secondary models could not be generated for *C. sakazakii* in reconstituted SB-PIF or for *S. enterica* in either of the reconstituted PIF formulations due to non-linearity.

### 3.6 Population dynamics of *C. sakazakii* and *S. enterica* in stored reconstituted powdered infant formula (PIF)

**FIGURE 7:**
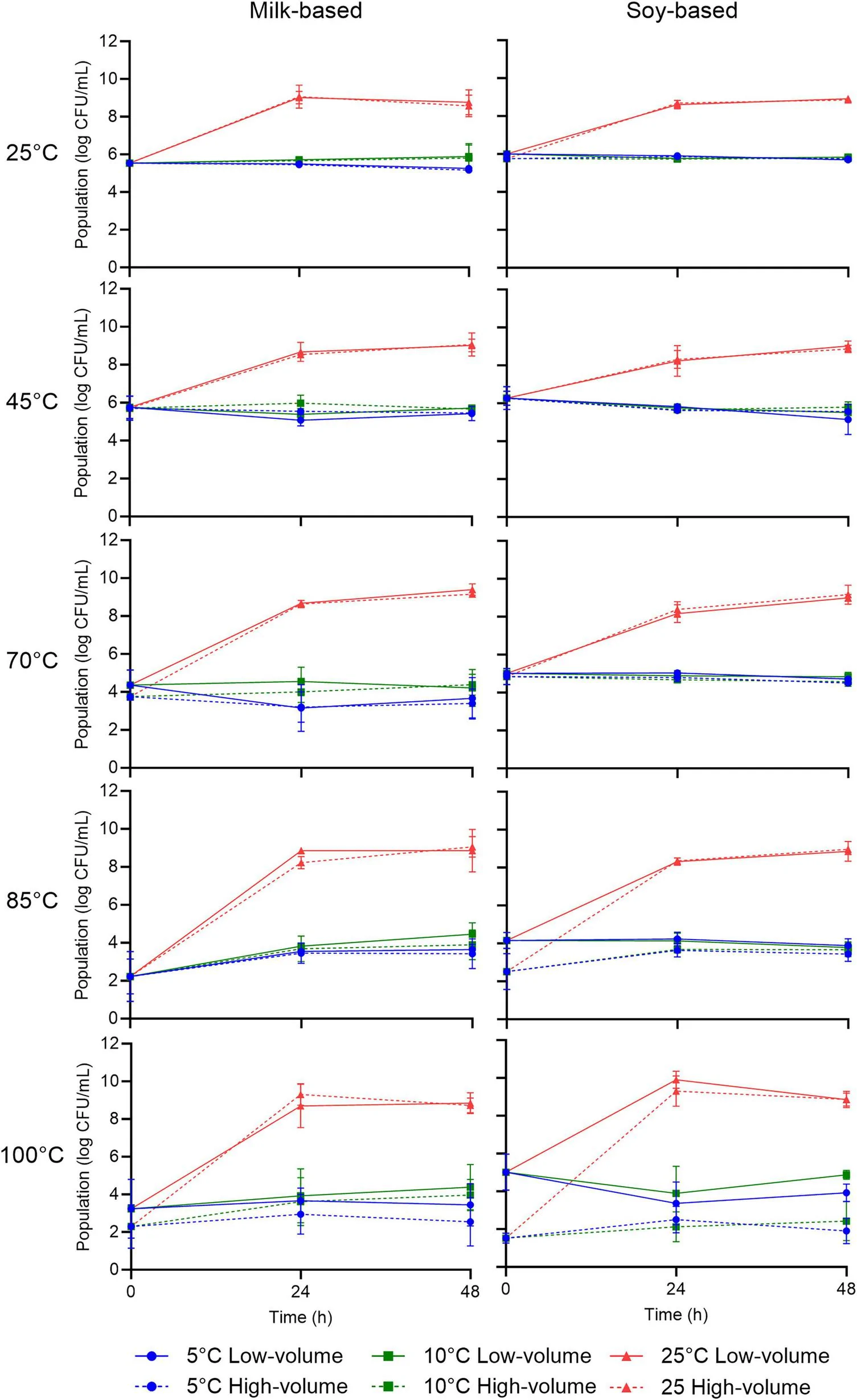
Population dynamics of *Cronobacter sakazakii* in a low-volume (LV) or high-volume (HV) of milk-based (MB) or soy-based (SB) powdered infant formula (PIF) reconstituted with an initial water temperature (*T*_*i*_) of 25, 45, 70, 85, or 100 °C and stored at 5, 10, or 25 °C for up to 48 h. Data are means ± standard deviations (*n* = 6).

**FIGURE 8:**
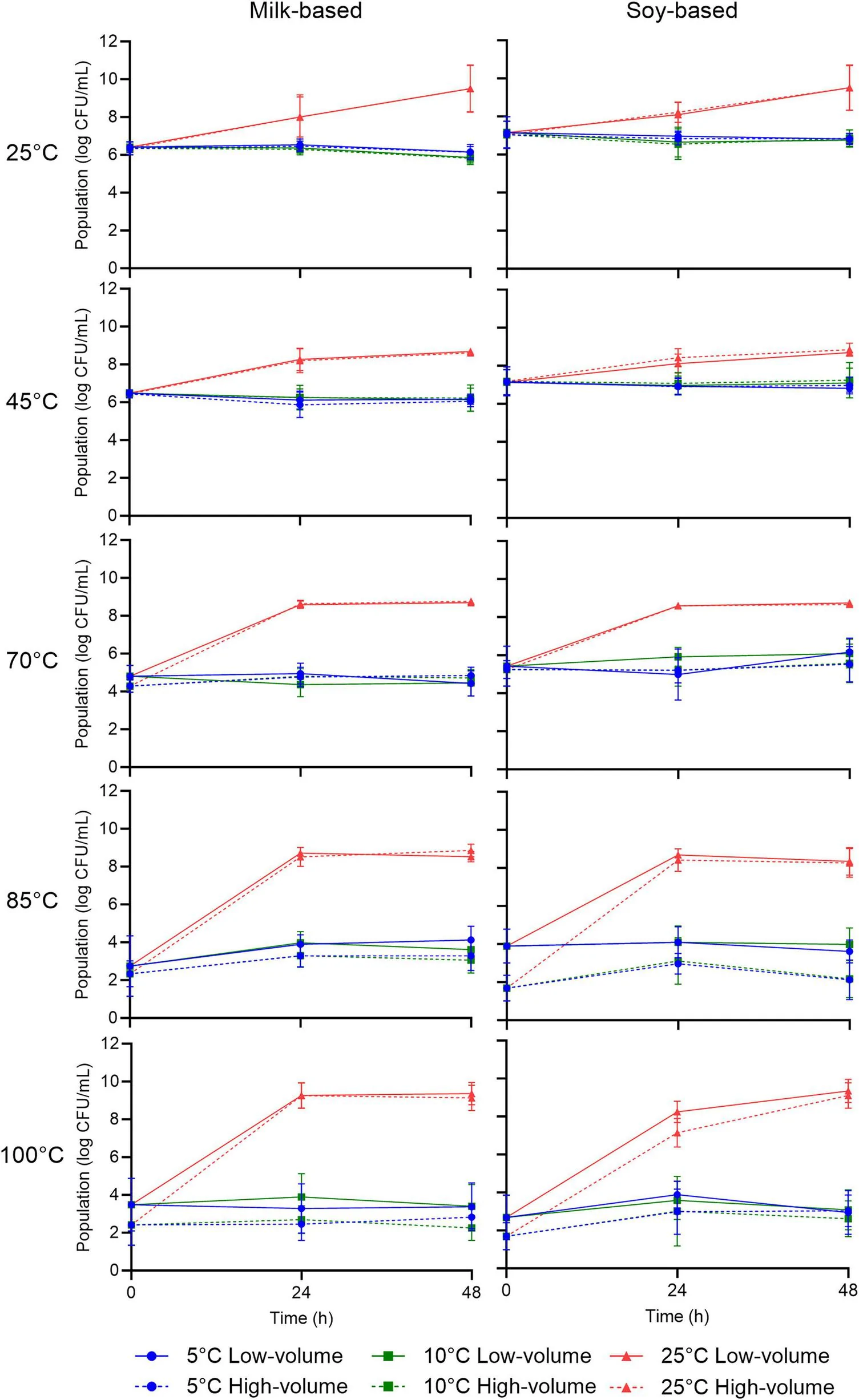
Population dynamics of *Salmonella enterica* in a low-volume (LV) or high-volume (HV) of milk-based (MB) or soy-based (SB) powdered infant formula (PIF) reconstituted with an initial water temperature (*T*_*i*_) of 25, 45, 70, 85, or 100 °C and stored at 5, 10, or 25 °C for up to 48 h. Data are means ± standard deviations (*n* = 6).

Storage temperature had a critical impact on both *C. sakazakii* and *S. enterica* populations in stored reconstituted PIF (Figures 7, 8, respectively). Regardless of the initial reconstitution water temperature (*T*_*i*_), regrowth of both pathogens occurred when samples were stored at 25 °C, regardless of PIF type, with all samples reaching populations of ≥ 8 log CFU/mL after 24 h. The greatest population increases were observed in samples reconstituted with 85 and 100 °C initial water temperature (increases of ≥ 6 log CFU/mL). Occasionally, samples stored at 5 or 10 °C showed slight growth (< 1 log CFU/mL), however populations in the majority of samples remained steady or displayed slight reductions after 48 h, regardless of pathogen, sample volume, or initial water reconstitution temperature.

## 4 Discussion

This study evaluated the effect of reconstitution water temperature and preparation volume on the inactivation, survival, and growth of *C. sakazakii* and *S. enterica* in MB- and SB-PIF. The reconstitution temperatures in this study included 25 °C to mimic home preparation using room temperature water as well as water at 45, 70, 85, or 100 °C. After reconstitution, PIF was passively cooled at ambient temperature (20–22 °C) for 240 min (4 h) as an extension beyond the recommended 2 h, followed by storage at 5, 10, or 25 °C to understand the effect of refrigeration and abuse temperatures on the potential survival or growth of the pathogens.

In this study, both *C. sakazakii* and *S. enterica* exhibited substantial differences in inactivation upon reconstitution of the PIF, with ≥ 4 log CFU/mL reductions achieved inconsistently when water at an initial temperature of 70 °C was used. The zero-time water temperatures across all samples ranged from 52.0 to 63.4 °C and 61.0–68.9 °C for LV and HV samples, respectively, indicating that home preparation temperatures would likely fall short of the 70 °C temperature recommended by WHO for home preparation for at-risk infants (WHO, 2007). Of the LV samples reconstituted with 70 °C water, only SB-PIF allowed for initial population reductions (within the first 5 min) of ≥ 4 log CFU/mL for both pathogens; however, populations increased after only a minimal cooling time ( < 30 min). For the HV samples reconstituted with 70 °C water, *C. sakazakii* was initially inactivated ≥ 4 log CFU/mL in both PIF formulations, however only *S. enterica* in MB-PIF achieved the same reduction. The HV samples were also found to result in less pathogen outgrowth during passive cooling, regardless of the formula type.

It should be noted that initial pathogen populations in the reconstituted PIF ranged from approximately 5–7 log CFU/mL across trials; given the limit of enumeration (1.39 log CFU/mL), the maximum reduction measurable in any individual trial was not always > 4 log CFU/mL. Reported reductions in trials where populations were reduced to the limit of enumeration should therefore be interpreted as lower-bound estimates, since the true extent of inactivation may exceed what could be measured by this assay. The relatively high inoculum level used in this study (ca. 7–8 log CFU/g dry PIF) was necessary to keep several log reductions measurable above the limit of enumeration across the range of reconstitution temperatures tested; however, this inoculum level is substantially higher than the contamination levels expected in naturally contaminated dry PIF, which are typically much lower, and this represents a limitation in directly extrapolating these findings to naturally occurring contamination scenarios. Highly variable zero-time temperatures, along with variable pathogen outgrowth during cooling of reconstituted PIF, have been noted by other studies (Edelson-Mammel and Buchanan, 2004; Losio et al., 2018; Beary et al., 2025). One study noted that zero-time temperatures were 10–13 °C lower when reconstituting PIF inoculated with approximately 1–1.5 log CFU/mL of *C. sakazakii* or *S. enterica* using water at an initial temperature of 70 °C in plastic bottles (Losio et al., 2018). While 1 out of 3 samples reconstituted at 70 °C were initially below the detection limit of 1 log CFU/mL for both pathogens, the pathogens were detected in all three samples after 2 h of storage at room temperature. Other studies also noted substantial differences between initial water temperatures and the zero-time temperature of the reconstituted PIF (Edelson-Mammel and Buchanan, 2004; Beary et al., 2025). For example, one study noted a zero-time temperature of approximately 62 °C when using water at an initial temperature of 70 °C for reconstitution of PIF (Edelson-Mammel and Buchanan, 2004). Another study observed up to 20 °C differences in the initial water temperature and the zero-time temperature when adding 2 oz (59 mL) of water to PIF in 4 oz (118 mL) glass bottles (Beary et al., 2025).

More consistent reductions in *C. sakazakii* and *S. enterica* populations were observed when water at an initial temperature of 85 or 100 °C was used in this study. The effect of the preparation volume was most evident for *C. sakazakii* in SB-PIF where reconstitution water temperatures of 85 and 100 °C resulted in no outgrowth during the 240 min (4 h) cooling period in the HV samples, however populations in the LV samples increased by approximately 3–4 log CFU/mL in the same amount of time. This is contrary to previously published literature showing that initial water temperatures of ≥ 80 °C consistently result in at least 4 log CFU/mL inactivation of both *S. enterica* and *C. sakazakii* in reconstituted PIF with no subsequent outgrowth (Losio et al., 2018). One theory proposed is that higher temperatures have a negative effect on PIF hydration, potentially caused by clumping which could protect pathogens from inactivation (Podolak and Black, 2017); however, no clumping was observed during reconstitution in this study. In addition, this study used PIF inoculated via atomization, followed by a 1 mo. equilibration period, which may have allowed higher pathogen heat tolerance than previously documented.

An alternative explanation for the observed recovery of *C. sakazakii* is regrowth of background microbiota misidentified as the target pathogen. However, background microbiota in the uninoculated PIF was quantified at ∼1 log CFU/g for both formulations, well below the levels of *C. sakazakii* recovery reported in this study, and colony identity was confirmed on a selective/differential chromogenic medium when morphology was ambiguous, making this an unlikely explanation for the recovery observed. It is also possible that the 1-month dry equilibration period altered the physiological state or thermal tolerance of the inoculated cells relative to freshly cultured cells, which was not independently assessed in this study. Additionally, LV and HV samples were reconstituted in differently sized bottles (125 and 500-mL, respectively); differences in bottle geometry, headspace, and surface-area-to-volume ratio between these containers may have influenced cooling rate independently of sample volume, however this was not addressed in the current study.

The dynamic log-linear model fit the experimental data with adequate RMSE values across all conditions, confirming its suitability for describing thermal inactivation under non-isothermal conditions typical of home preparation of reconstituted PIF. *S. enterica* inactivation kinetics appeared to be more linear for the LV samples than for the HV samples. The mean *S. enterica k*_*max*_ values did not correlate well with reconstitution water temperature, and thus secondary models were not developed. Linear regression of the square root of *k*_*max*_ against *T*_0_ yielded *r*^2^ values of 0.68 and 0.07 for LV and HV samples, respectively, in MB-PIF, and 0.06 and 0.11 for LV and HV samples, respectively, in SB-PIF. A secondary model could also not be generated for *C. sakazakii* in reconstituted SB-PIF (*r*^2^ = 0.01 and < 0.001 for LV and HV samples, respectively). Secondary models were successfully generated for *C. sakazakii* in MB-PIF for both preparation volumes. A strong linear relationship (*r*^2^ = 0.83 and 0.85 for LV and HV samples, respectively) between the initial water temperature and the maximum inactivation rate was observed in MB-PIF, providing a quantitative framework for predicting pathogen reduction as a function of reconstitution water temperature.

This predictive capacity is valuable for risk assessment modeling and for establishing science-based temperature thresholds. However, it should be noted that because D and z were estimated jointly from time points collected prior to reaching the limit of enumeration, trials in which the population was reduced below this limit within 1–3 min necessarily had fewer informative points available for fitting. In these cases, multiple D/z combinations can yield comparably low residual error, so the reported *k*_*max*_ values for the fastest-inactivating trials should be interpreted as consistent with rapid, near-complete inactivation rather than as precisely resolved rate estimates.

The storage experiments in this study revealed that both pathogens demonstrated rapid proliferation when stored at room temperature, including in samples where populations had been significantly reduced by high-temperature water reconstitution. Regrowth to populations ≥ 8 log CFU/mL was observed after 24 h at 25 °C, regardless of initial water reconstitution temperature. Even samples initially reconstituted at 100 °C, where populations had been reduced to < 1.39 log CFU/mL and no growth was observed during passive cooling, exhibited regrowth when subjected to 25 °C storage for even 24 h. Occasional minimal ( < 1 log CFU/mL) growth also occurred during storage at 10 °C. Only storage at 5 °C effectively prevented pathogen growth, with populations remaining stable over 48 h. It should be noted that population increases observed during passive cooling and storage may reflect resuscitation and repair of sublethally injured cells in addition to, or instead of, true multiplication. The enumeration method used in this study did not distinguish between these mechanisms, and this represents a limitation in interpreting the regrowth reported. These findings align with previous studies documenting rapid *C. sakazakii* recovery following mild heat treatments (Osaili et al., 2009b; Parra-Flores et al., 2015) and emphasize that temperature control during storage is as critical as reconstitution water temperature for ensuring microbiological safety.

Results from this study have determined that PIF reconstitution water temperature is essential to control *C. sakazakii* and *S. enterica*. The use of water ≤ 70 °C may permit the survival of low but epidemiologically significant pathogen populations, particularly in SB-PIF formulations. Initial water temperatures of 85 and 100 °C, especially for MB-PIF, resulted in inactivation of both pathogens and also limited outgrowth during passive cooling of reconstituted PIF. However, a limitation of this study is that nutrient degradation was not evaluated. Heating water to ≥ 70 °C may reduce concentrations of heat-labile vitamins (such as vitamin C, folate, and B-complex vitamins) and may negatively affect bioactive proteins, prebiotics, and probiotics when present (Agostoni et al., 2004). Such losses could influence the nutritional value of reconstituted PIF if hot water is used consistently for all feedings. A recent analysis of manufacturer labeling emphasized the need to balance microbial safety with nutrient preservation when specifying PIF reconstitution instructions (Beary et al., 2025). Future research could quantify nutrient retention alongside microbial safety outcomes to establish optimized preparation parameters that safeguard both infant health and PIF quality.

## Data Availability

The original contributions presented in the study are included in the article/Supplementary material, further inquiries can be directed to the corresponding author.
