# Supplementary material for: Inactivation kinetics of Cronobacter sakazakii and Salmonella enterica in reconstituted powdered infant formula and survival during subsequent storage
Source: Front Microbiol. 2026 Aug 27;17:1939855. doi: 10.3389/fmicb.2026.1939855 (PMC13558135; doi:10.3389/fmicb.2026.1939855)
Supplement: Supplementary file 1 [file Table_1.docx]

**Figure S1.** Cooling temperature profiles of low-volume (LV; solid lines, square data points) and high-volume (HV; dotted lines, circle data points) reconstituted milk-based (MB) and soy-based (SB) powdered infant formula (PIF) at room temperature (20-22°C) for 60 min when the initial temperature of the water (*T_i_*) was 25, 45, 70, 85, or 100°C. Data are means ± standard deviations (n=12)**.**
